# The Diagnostic Value of Pulsar Perimetry, Optical Coherence Tomography, and Optical Coherence Tomography Angiography in Pre-Perimetric and Perimetric Glaucoma

**DOI:** 10.3390/jcm10245825

**Published:** 2021-12-13

**Authors:** Hung-Chih Chen, Michael Chia-Yen Chou, Ming-Tsung Lee, Chia-Yi Lee, Che-Ning Yang, Chin-Hsin Liu, Shih-Chun Chao

**Affiliations:** 1 Department of Ophthalmology, Show Chwan Memorial Hospital, Changhua 500, Taiwan; b101098004@tmu.edu.tw (H.-C.C.); mike_chou328@hotmail.com (M.C.-Y.C.); ao6u.3msn@hotmail.com (C.-Y.L.); 2 Preparatory Office of National Center for Geriatrics and Welfare Research, National Health Research Institutes, Yunlin 632, Taiwan; lee6717kimo@yahoo.com.tw; 3 Department of Nursing, Hungkuang University, Taichung 433, Taiwan; 4 Department of Medicine, National Taiwan University, Taipei 100, Taiwan; jamesyang860817@gmail.com; 5 Department of Ophthalmology, Yonghe Cardinal Tien Hospital, New Taipei City 234, Taiwan; 6 Department of Optometry, Central Taiwan University of Science and Technology, Taichung 406, Taiwan; 7 Department of Optometry, Yuanpei University of Medical Technology, Hsinchu 300, Taiwan

**Keywords:** pulsar perimetry, optical coherence tomography angiography, pre-perimetric glaucoma

## Abstract

The purpose of this article is to investigate the diagnostic value of Pulsar perimetry (PP), optical coherence tomography (OCT), and optical coherence tomography angiography (OCTA) in pre-perimetric glaucoma (PPG) and perimetric glaucoma (PG). This retrospective cross-sectional study included 202 eyes (145 eyes in the control group, 40 eyes in the PPG group, and 17 eyes in the PG group) from 105 subjects. The results were analyzed by paired t-tests and Wilcoxon signed-rank test. The area under the curve (AUC), sensitivity, and specificity were used to evaluate the diagnostic accuracy. Pearson correlation was used to investigate the relationships of each parameter. The most sensitive parameters for differentiating the control group from the PPG group by using Pulsar, OCT, and OCTA were square loss variance of PP (AUC = 0.673, *p* < 0.001), superior ganglion cell complex thickness (AUC = 0.860, *p* < 0.001), and superior-hemi retina thickness (AUC = 0.817, *p* < 0.001). In the PG group, the most sensitive parameters were mean defect of PP (AUC = 0.885, *p* < 0.001), whole image of ganglion cell complex thickness (AUC = 0.847, *p* < 0.001), and perifoveal retina thickness (AUC = 0.833, *p* < 0.001). The mean defect of PP was significantly correlated with vascular parameters (radial peripapillary capillary (RPC), *p* = 0.008; vessel density of macular superficial vascular complex (VDms), *p* = 0.001; vessel density of macular deep vascular complex (VDmd), *p* = 0.002). In conclusion, structural measurements using OCT were more sensitive than vascular measurements of OCTA and functional measurements of PP for PPG, while PP was more sensitive than the structural and vascular measurements for PG. The mean defect of PP was also shown to be highly correlated with the reduction of vessel density.

## 1. Introduction

Glaucoma, an irreversible and progressive optic neuropathy, is the second-leading cause of blindness in the world [1]. The true prevalence of glaucoma is often underestimated, since more than half of the patients with glaucoma are undiagnosed [2]. Improving the diagnostic capability of tests for glaucoma is needed, since this disease is mostly asymptomatic until the late stage, where irreversible and often severe visual deficits occur [3].

There may be structural or functional abnormalities in early glaucoma, including changes in the optic disc, the retinal nerve fiber layer (RNFL), the macular retinal ganglion cell complex (GCC), or visual field [4,5,6,7,8]. Optical coherence tomography (OCT) and optical coherence tomography angiography (OCTA) are used to evaluate structural and capillary abnormalities, and standard automated perimetry (SAP) is used to detect functional defects. Due to the various diagnostic capabilities of the above-mentioned instruments at different stages of glaucoma, associated researches and utilities in diagnosing and monitoring glaucoma are still developing.

SAP is currently accepted as the gold standard of glaucoma diagnosis. However, in patients with pre-perimetric glaucoma (PPG), SAP does not detect visual field defects until about 30–50% of retinal ganglion cell (RGC) injury [9,10], which pose a diagnostic dilemma. Its detectability of early glaucomatous visual field damage has been reported to be slightly inferior to that of the structural measurements by optical coherence tomography (OCT) [11,12]. On the other hand, functional measurements from non-conventional perimetry, such as Pulsar perimetry (PP), can assist to diagnose early glaucoma [13,14,15,16,17,18]. PP was initially reported by González-Hernandez et al. in the 2000s [13,19,20,21]; it can detect temporal and spatial contrast sensitivity functions simultaneously [15]. PP is considered to be an important instrument in evaluating early glaucoma, especially in the pre-perimetric stage. Additionally, OCTA has been used to evaluate the vessel density changes in PPG [22,23,24,25,26,27,28,29,30,31], while its diagnostic value has not been completely established.

Due to the limitations of current instruments, it is important to evaluate the full dimensions of parameters, including structural, functional, and vascular changes associated with the detection of early glaucoma. The aim of this study is to compare the diagnostic value of Pulsar perimetry with parameters of OCT and OCTA for pre-perimetric and perimetric glaucoma (PG) and to investigate the correlation of the above parameters in the patients of PPG.

## 2. Materials and Methods

### 2.1. Study Design, Inclusion, and Exclusion Criteria

This retrospective cross-sectional study was reviewed and approved by the ethics committee and Institutional Review Board (IRB) of the Cardinal Tien Hospital (CTH-106-3-6-035) in compliance with the tenets of the Helsinki’s Declaration. All study subjects were provided written informed consent. Inclusion criteria consisted of patients who received a comprehensive basic and advanced ophthalmic examinations during the acceptance period. The exclusion criteria were as follows: best corrected visual acuity worse than 20/40, spherical equivalent exceeding the range of −8.00 to +5.00 diopter, intraocular pressure more than 21 mmHg, underlying ophthalmic diseases such as retinal degeneration, neurological disorder, macular disease, and severe to end-stage glaucoma.

### 2.2. Study Participants

This study included 202 eyes (145 eyes in the control group, 40 eyes in the PPG group, and 17 eyes in the PG group) from 105 subjects who visited the department of ophthalmology at Yonghe Cardinal Tien Hospital, New Taipei City, Taiwan from September 2017 to December 2017. All subjects underwent comprehensive ophthalmologic examinations including best corrected visual acuity, intraocular pressure measurement, refractive error measurement through autorefraction, central corneal thickness measurement, slit-lamp, gonioscope, and fundus examination performed by a glaucoma specialist, as well as advanced exams including SAP, PP, OCT and OCTA. Subjects would receive a brief training session and practice all the instruments before initiating the examination. Patients were classified as PPG in the presence of a normal SAP test with mean defect (MD) < 2 dB, normal anterior segment, and an open angle in gonioscope, but presenting with glaucomatous optic neuropathy including optic nerve rim defect (notching or localized thinning), optic disc hemorrhage, or nerve fiber layer defects; while patients were classified as PG if they showed glaucomatous optic neuropathy with abnormal SAP results (MD > 2 dB) corresponding to a glaucomatous visual field. SAP and PP results were considered reliable if the fixation loss was <20%, the false positive rate was <15%, and the false negative rate was <33%. The diagnosis of glaucomatous neuropathy was determined through a fundus examination performed by a glaucoma specialist.

### 2.3. Examination Instruments

#### 2.3.1. Functional Parameters: Standard Automated Perimetry (SAP) and Pulsar Perimetry (PP)

SAP was performed by Octopus 600, Haag-Streit AG, Switzerland. The principle is to detect the threshold of differential light sensitivity (white-on-white). Technical specifications are 30° peripheral range, dynamic range around 35 dB, 0.43° (Goldmann III), G,32.24-2 programs. The recorded parameters were mean sensitivity (MS), mean defect (MD), and square loss variance (sLV).

PP was performed by Octopus 600, T30W, Haag-Streit AG, Switzerland. The principle is to detect the threshold of flicker, contrast, and spatial resolution. Threshold sensitivity is expressed in spatial resolution contrast units (src). The specifications are 30° peripheral range, about 35 src dynamic range, and GP, 32p programs. The recorded parameters are the same as those of SAP (MS, MD, sLV) as are the reliability criteria.

#### 2.3.2. Structural Parameters: Optical Coherence Tomography (OCT)

Spectrum-domain OCT (SD-OCT) was performed by RTVue XR, Avanti, USA. Commercial specifications include 840 ± 10 nm wavelength, axial A-scan rate 70 kHz, 5 µm optical resolution depth in tissue (3 µm digital depth of image sampling rate), 2 to 12 mm transverse scan range, and 3 mm scan depth. Combo scan patterns of RNFL, optic nerve head (ONH), and GCC were captured.

#### 2.3.3. Vascular Parameters: Optical Coherence Tomography Angiography (OCTA)

OCTA was performed by AngioVue OCTA, Optovue, CA, USA. Split-spectrum amplitude decorrelation angiography (SSADA) is utilized to simulate real circulation by detecting red blood cell motion in the vessels obtained from sequential B-scan at a single cross-section of the target tissue. Multilayered widefield views of the retinal vasculature were obtained by enface visualization and angiomontage. The vessel density is defined by the ratio of the total vessel occupying an area in the expected measured region (%) via the AngioAnalytic software.

**Figure 1:**
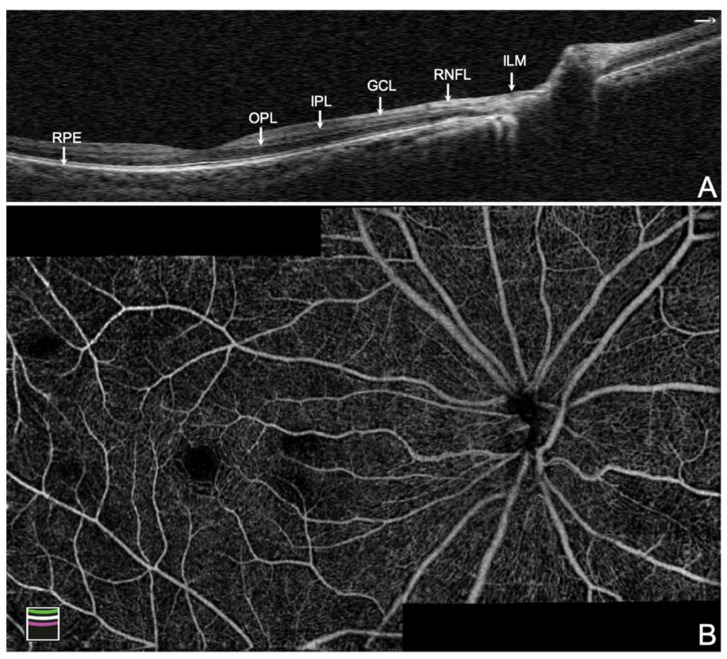
Composition of the retinal vascular plexus. (**A**) The cross-section of wide-field optical coherence tomography of disc and macula. (**B**) The montage mode of 6 × 6 mm^2^ optical coherence tomography angiography of disc and macula algorithm of a normal eye. SVC = superficial vascular complex (plexus of RNFL + GCL + IPL); RNFL = retinal nerve fiber layer; GCL = ganglion cell layer; IPL = inner plexiform layer; DVC = deep vascular complex (plexus of IPL to OPL); OPL = outer plexiform layer; ILM = internal limiting membrane; RPE = retinal pigmented epithelium.

Three algorithms of vessel density were analyzed in our study: (1) radial peripapillary capillary (RPC), (2) superficial vascular complex (SVC), (3) deep vascular complex (DVC). The vessel density of RPC was measured in the slab extending from the internal limiting membrane (ILM) to the RNFL posterior boundary in a 4.5 × 4.5 mm^2^ field of view fixating at the ONH by AngioVue disc mode. Peripapillary RPC was calculated within a region encompassing a 750 μm wide elliptical annulus extending from the optic disc boundary, and it was divided into different regions. The vessel density of SVC in the macular area (VDms) was calculated in the slab extending from the ILM to the posterior border of the inner plexiform layer (IPL), while the vessel density of DVC in the macula (VDmd) was calculated in the slab extending from posterior border of the IPL to the posterior border of the outer plexiform layer (OPL) in a 6 × 6 mm^2^ field of view fixating at the fovea by AngioVue macular mode (Figure 1). The capillary plexus projections are slightly offset compared with the corresponding retinal layers on structural OCT due to the commercial settings of the machine. Whole image vessel density was calculated in the 6 × 6 mm^2^ scan; foveal vessel density was measured in the inner 1 mm diameter ring; the parafoveal area was measured between the inner 1 mm and the outer 3 mm diameter ring; and the perifoveal area was measured between the 3 mm and the outer 6 mm diameter ring. All of the images were centered on the fovea. Poor quality images, including images with (1) a signal strength index (SSI) less than 48, (2) poor clarity, (3) residual motion artifacts visible as irregular vessel pattern or obscured disc boundary on the enface angiogram, (4) local weak signal (due to vitreous opacity, floater, etc.), and (5) segmentation errors, were excluded.

### 2.4. Statistical Analysis

The results were analyzed by paired t-tests and Wilcoxon signed-rank test. The area under the curve (AUC), sensitivity, and specificity, were used to evaluate diagnostic accuracy. The best cut-off parameter for discerning between the control and study groups was decided by the highest AUC based on receiver operating characteristic analysis. The sensitivities for target specificities were calculated, and the method of Delong was used to compare the sensitivity and AUC of different parameters. Pearson correlation was used to investigate the relationship between functional and structural glaucomatous measurements and peripapillary and macular capillary densities. All statistical analyses were performed using IBM SPSS Statistics for Windows, version 24.0 (IBM Corp., Armonk, NY, USA) and MedCalc version 10.1.3.0 software (Ostend, Belgium). Since multiple testing might increase the false positive rate, we defined the statistical significance threshold as *p* value less than 0.01.

## 3. Results

Table 1 showed the demographic data of normal participants, PPG patients, and PG patients. We revealed a significant difference in measurements with SAP (MS, MD, and sLV) between the control and PG groups, while there was no significant difference between the control and PPG groups. This was compatible with the definition of pre-perimetric glaucoma.

Table 2 showed the results of each parameter obtained from Pulsar perimetry, OCT, and OCTA in normal participants, PPG patients, and PG patients. We observed significant differences in the greatest number of parameters between the control and PPG group (A–B) and between the control and PG group (A–C). This confirms the ability of these instruments in detecting corresponding functional and structural glaucomatous change and difference in capillary perfusion between the control and glaucoma group.

Table 3 revealed that the most sensitive parameters for discerning the control group from the PPG group using Pulsar, OCT, and OCTA (disc and macular algorithms) were square loss variance of PP (AUC = 0.673, *p* < 0.001), superior ganglion cell complex thickness (AUC = 0.860, *p* < 0.001), whole image of radial peripapillary capillary density (AUC = 0.791, *p* < 0.001), and superior-hemi retina thickness (AUC = 0.817, *p* < 0.001), respectively. Generally, the AUC of structural parameters were better than Pulsar and measurements of vessel density by OCTA.

Table 4 revealed that the most sensitive parameters for discerning the control group from the PG group using Pulsar, OCT, and OCTA (disc and macular algorithms) were the mean defect of PP (AUC = 0.885, *p* < 0.001), whole image of ganglion cell complex thickness (AUC = 0.847, *p* < 0.001), superior-hemi RNFL thickness (AUC= 0.856, *p* < 0.001), and perifoveal retina thickness (AUC = 0.833, *p* < 0.001), respectively.

**Figure 2:**
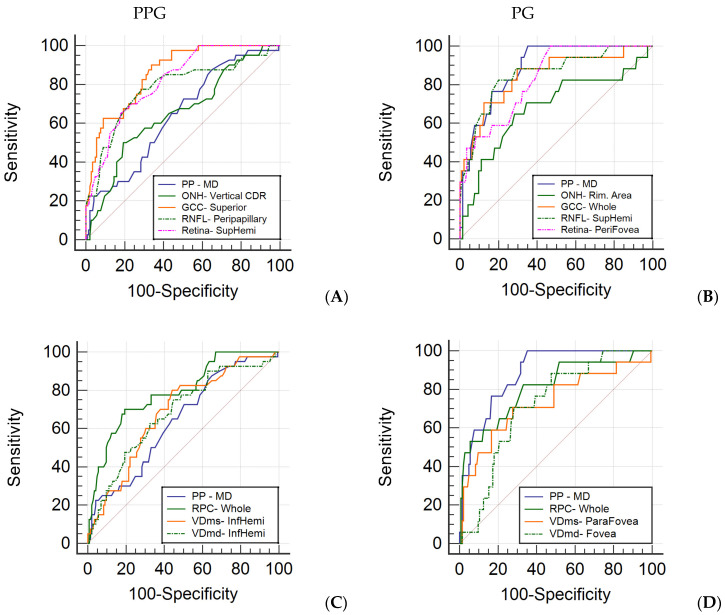
Receiver operating characteristic curve of most sensitive parameters in each category. Area under the curve (AUC) of the most sensitive parameters from Pulsar perimetry and structural parameters of OCT and OCTA in PPG (**A**) and PG (**B**). Area under the curve (AUC) of the most sensitive parameters from Pulsar perimetry and capillary parameters of OCTA in PPG (**C**) and PG (**D**). PP = Pulsar perimetry; MD = mean defect; ONH = optic nerve head; CDR = cup–disc ratio; GCC = ganglion cell complex; RNFL = retinal nerve fiber layer; RPC = radial peripapillary capillary; VDms = vessel density of macular superficial vascular complex; VDmd = vessel density of macular deep vascular complex; SupHemi = superior-hemi; InfHemi = inferior-hemi.

We also presented the result of the best cut-off value, sensitivity, specificity, and sensitivity at 80% and 90% specificity between the control group and the PPG group and between the control group and the PG group in Table 3 and Table 4. The diagram of AUC illustrates the relationship of PP versus the structural parameters and capillary parameters in each group (Figure 2).

Generalized parameters obtained from the PPG group were investigated in correlation with one another. The GCC, RNFL, and retina thickness showed a better correlation with structural parameters than the rim area of ONH. RPC, VDms, and VDmd also revealed a fair correlation with vascular parameters. We revealed that PP was significantly correlated with vascular parameters (RPC, *p* = 0.008; VDms, *p* = 0.001; VDmd, *p* = 0.002). SAP was significantly correlated with GCC (*p* = 0.008) and VDms (*p* = 0.002) (Table 5).

## 4. Discussion

In our study, Pulsar perimetry (PP) presented great value in diagnosing perimetric glaucoma (PG), while optical coherence tomography (OCT) presented great value in diagnosing pre-perimetric glaucoma (PPG). The satisfactory correlation among functional, structural, and vascular parameters was revealed in PPG. Combining different parameters of varying diagnostic strength improved the early detection of glaucoma and helped establish appropriate monitoring strategies to prevent severe visual complications resulting from undiagnosed glaucoma.

Untreated PPG has been shown to progress gradually, with a progression probability at 5 years of 39% by structural criteria and 5% by functional criteria [32]. Thus, the diagnostic capability of PPG is vital in the early diagnosis and prevention of glaucoma. Non-conventional perimetric exams have proven to be a useful method to discriminate PPG from normal subjects; Pulsar perimetry was reported to have a wider area under curve (AUC) = 0.733 [15] and higher sensitivity in comparison to the standard automated perimetry (SAP) in detecting loss of visual field in early glaucoma [15,33,34]. We reported the mean defect (MD) of PP with the sensitivity at the best cut-off value = 87.5% and the square loss variance (sLV) of PP with AUC = 0.673 (*p* < 0.001) (Table 3). OCT was used to evaluate and monitor the change of the nerve fiber structure, with the reported AUC = 0.527 to 0.938 in the previous studies [35,36]. We revealed that the most sensitive parameters of OCT were superior ganglion cell complex (GCC) thickness with AUC = 0.860 (*p* < 0.001) and the sensitivity at the best cut-off value = 90% (Table 3). With the development of optical coherence tomography angiography (OCTA), the perfusion of the optic disc and macula was considered as an early indicator of glaucomatous change, with a reported AUC of macular vascular density = 0.88 [23] and a significantly decreased circumpapillary vascular density [22]. We reported that the most sensitive parameters of OCTA was the whole image of radial peripapillary capillary (RPC) density with AUC = 0.791 (*p* < 0.001) and sensitivity at the best cut-off value = 70% (Table 3). The diagnostic capability for PPG with the above devices revealed that structural measurements appeared to be more accurate and sensitive than functional and vascular measurements. This may be explained by the fact that PPG is routinely diagnosed by clinical structural assessment of the optic disc and consequent peripapillary nerve change based on the mechanism of glaucomatous pathogenesis [37].

In perimetric glaucoma, we reported that the most accurate functional parameter was MD of PP with AUC = 0.885 (*p* < 0.001) and sensitivity at the best cut-off value = 100%, while the structural parameter using superior-hemi retinal nerve fiber layer (RNFL) thickness had an AUC = 0.856 (*p* < 0.001) and sensitivity at the best cut-off value = 82.4% (Table 4). We also reported that the most accurate vascular parameter to evaluate PG was whole image of RPC density with AUC = 0.809 (*p* < 0.001) and sensitivity at the best cut-off value = 82.4% (Table 4). The diagnostic capability of PG revealed that the functional measurement had better results than structural and perfusional measurements. The severity of PG can be categorized using MD of SAP according to the Hodapp–Parrish–Anderson glaucoma staging system. In this system, early visual field (VF) defects are characterized by an MD up to 6 dB, moderate VF defects are characterized by a MD ranging from 6 to 12 dB, and severe VF defects are characterized by an MD worse than 12 dB [38]. On the other hand, the structural parameters have a floor effect in severe glaucoma; OCT parameters reach a base level beyond which little change is seen with increasing severity of glaucoma [39]. As a result, functional assessment may be more sensitive than structural assessment.

In previous literature discussing the change of vessel density in glaucomatous eyes, the reduction in vasculature is reported to be more pronounced in the RPC slab compared to the deep retinal slabs [40], and it is also more pronounced in the superficial retinal slab compared to the deep retinal slabs [41]. We reported the same result that AUC and sensitivity at the best cut-off value of RPC was better than VDms and VDmd in PPG and PG groups (Figure 2). We also compared the diagnostic capability of Pulsar perimetry with OCTA and OCT measurements, which showed PP having better performance in the PG group and worse performance in the PPG group (Figure 2). Understanding the relationship of visual field defects with RNFL or GCC thinning and vessel density reduction helps the clinicians develop strategies to detect glaucoma in the earliest stages. Hirasawa et al. reported that the agreement between structural and specific functional measurements in patients with PPG and early glaucoma was poor; however, both groups were able to be diagnosed with 100% sensitivity using either structural or specific functional measurements [18]. We revealed that PP was more significantly correlated with vessel density of RPC and macula than SAP in pre-perimetric glaucoma eyes (Table 5), with high sensitivity at the best cut-off value in both PPG and PG groups (Table 3 and Table 4). Previous literatures found reduced peripapillary vessel density and RNFL thickness in the hemiretina corresponding to the perimetrically intact hemifield in patients with PG [42,43,44]; even OCTA changes may precede RNFL changes in some sectors [44]. Due to the variations in the initial presentation of glaucoma and different diagnostic strength of examination instruments, a combination of measurements using Pulsar perimetry, OCT, and OCTA should be recommended to reliably diagnose glaucoma in an extremely early stage.

There are some limitations in our study. Firstly, the sample size of patients with perimetric glaucoma is limited; the distribution of sample size between the control and study groups is also unequal. Furthermore, the composition percentage of glaucoma type (i.e., open-angle glaucoma and normal tension glaucoma) in the PPG and PG groups may be different. Secondly, early glaucoma may have been missed or underestimated with SAP using a 6 degrees grid, such as the 24-2 algorithm [45]. More detailed testing (i.e., 10-2 algorithm) may aid in resolving this issue and enable the early detection of glaucoma. Thirdly, raw data from OCTA imaging may have been interfered by flow projection artifacts [46]; the projection-resolved OCTA (PR-OCTA) algorithm was reported to effectively remove the projection artifact and display more accurate vessel density [46,47,48]. Further studies are warranted to confirm our results.

## 5. Conclusions

Structural measurements were shown to be more sensitive than functional measurements performed by Pulsar perimetry and vascular measurements from OCTA for detecting PPG. On the other hand, Pulsar perimetry was more sensitive than the structural and vascular measurements gathered from OCT and OCTA for diagnosing PG. PP was highly correlated with the reduction of vessel density in RPC and macula. Utilizing a combination of different devices is advised to enable a more sensitive diagnosis of early-stage glaucoma. More longitudinal and randomized controlled studies are required for improving the diagnostic accuracy.

## Figures and Tables

**Table 1:** Demographics of the subjects of control and study groups.

Parameters	(A) Control (*n* = 145)		(B) PPG (*n* = 40)		(C) PG (*n* = 17)		*p* Value		
	Mean	SD	Mean	SD	Mean	SD	(A)–(B)	(A)–(C)	(B)–(C)
Age (years)	41.2	9.6	46.2	8.1	49.8	9.1	0.002 *	0.002 *	0.215
Sex (M:F)	26:119		9:31		1:16		0.514	0.310	0.253
VA (LogMAR)	0.036	0.044	0.026	0.032	0.041	0.036	0.203	0.602	0.212
IOP (mmHg)	15.0	3.2	14.3	3.3	14.5	3.0	0.216	0.491	0.876
CCT (μm)	551.8	41.9	541.7	58.2	542.9	44.6	0.220	0.450	0.930
SE (D)	−372.9	298.7	−506.3	359.2	−423.5	389.4	0.020	0.537	0.372
SAP MS (dB)	28.3	2.0	28.0	1.2	24.1	2.8	0.255	<0.001 *	<0.001 *
SAP MD (dB)	−0.6	1.3	−0.2	1.1	3.1	2.5	0.058	<0.001 *	<0.001 *
SAP sLV (dB)	1.6	0.6	1.7	0.5	3.0	0.8	0.700	<0.001 *	<0.001 *

PPG = pre-perimetric glaucoma; PG = perimetric glaucoma; SD = standard deviation; VA= visual acuity; LogMAR = logarithm of the minimum angle of resolution; IOP = intraocular pressure; CCT = central corneal thickness; SE = spherical equivalent; D = diopter; SAP = standard automated perimetry; MS = mean sensitivity; dB = decibel; MD = mean defect; sLV = square loss variance. * statistically significant (*p* < 0.01).

**Table 2:** Comparison of each parameter measured with Pulsar perimetry, OCT, and OCTA.

Parameters	(A) Control (*n* = 145)		(B) PPG (*n* = 40)		(C) PG (*n* = 17)		*p* Value		
	Mean	SD	Mean	SD	Mean	SD	(A)–(B)	(A)–(C)	(B)–(C)
**Pulsar perimetry**									
MD (src)	0.5	2.0	1.7	2.5	4.2	2.6	0.004 *	<0.001 *	<0.001 *
sLV (src)	1.9	0.7	2.3	0.7	2.8	0.8	0.001 *	<0.001 *	0.021
**OCT-ONH analysis**									
Vertical CDR	0.5	0.2	0.6	0.1	0.6	0.2	0.006 *	0.063	0.939
Horizontal CDR	0.6	0.2	0.7	0.2	0.7	0.1	0.156	0.190	0.774
Rim. Area (mm^2^)	1.4	0.4	1.3	0.3	1.2	0.4	0.133	0.034	0.337
**OCT-GCC thickness (μm)**									
Whole Image	98.0	5.2	91.0	5.0	89.3	7.5	<0.001 *	<0.001 *	0.285
Superior	98.1	5.3	90.5	4.8	92.3	6.8	<0.001 *	<0.001 *	0.247
Inferior	98.0	5.5	90.8	5.2	86.9	10.4	<0.001 *	<0.001 *	0.023
**OCTA (Disc)-RNFL thickness (μm)**									
Peripapillary	116.1	11.1	105.0	11.8	99.1	12.7	<0.001 *	<0.001 *	0.078
Superior-Hemi	116.5	12.8	104.6	12.6	99.7	10.9	<0.001 *	<0.001 *	0.186
Inferior-Hemi	115.6	11.3	105.4	12.6	98.6	18.6	<0.001 *	<0.001 *	0.059
**OCTA (Macular)-Retina thickness (μm)**									
Whole Image	282.8	10.8	269.9	9.6	267.9	11.2	<0.001 *	<0.001 *	0.522
Superior-Hemi	285.5	10.9	271.9	9.9	272.6	10.2	<0.001 *	<0.001 *	0.802
Inferior-Hemi	280.0	11.2	267.7	10.3	263.2	13.8	<0.001 *	<0.001 *	0.172
Fovea	247.9	19.1	241.4	15.8	246.7	18.3	0.049	0.804	0.318
ParaFovea	318.9	15.3	307.2	12.9	307.5	10.8	<0.001 *	0.003 *	0.928
PeriFovea	280.9	11.0	267.8	10.4	265.1	11.7	<0.001 *	<0.001 *	0.393
**OCTA (Disc)-RPC (%)**									
Whole Image	49.3	3.3	46.5	2.6	45.4	3.9	<0.001 *	<0.001 *	0.244
Inside Disc	51.2	5.9	50.4	5.6	48.5	7.7	0.432	0.073	0.269
Peripapillary	52.3	2.7	48.7	3.7	48.2	4.9	<0.001 *	<0.001 *	0.592
Superior-Hemi	52.4	3.0	48.8	4.2	48.3	5.0	<0.001 *	<0.001 *	0.666
Inferior-Hemi	52.2	2.8	48.5	3.8	48.0	6.1	<0.001 *	<0.001 *	0.568
**OCTA (Macular)-VDms (%)**									
Whole Image	47.7	3.4	45.4	3.8	44.1	5.1	<0.001 *	<0.001 *	0.202
Superior-Hemi	47.9	3.5	45.6	4.0	44.6	4.7	0.001 *	0.001 *	0.319
Inferior-Hemi	47.5	3.4	45.2	3.7	43.5	6.0	0.001 *	<0.001 *	0.119
Fovea	19.7	6.8	16.7	5.9	14.8	5.7	0.010	0.004 *	0.329
ParaFovea	48.8	4.6	46.5	4.9	44.5	5.8	0.007 *	<0.001 *	0.144
PeriFovea	48.6	3.5	46.2	3.8	45.0	5.0	<0.001 *	<0.001 *	0.244
**OCTA (Macular)-VDmd (%)**									
Whole Image	45.0	5.5	41.8	5.5	41.4	6.2	0.001 *	0.011	0.823
Superior-Hemi	45.1	5.6	42.1	5.8	42.2	6.2	0.003 *	0.046	0.945
Inferior-Hemi	44.9	5.7	41.4	5.5	40.6	6.5	0.001 *	0.003 *	0.606
Fovea	35.4	7.3	32.9	6.4	30.6	6.0	0.055	0.009 *	0.255
ParaFovea	51.7	4.7	49.3	4.8	49.8	5.8	0.004 *	0.109	0.726
PeriFovea	45.6	6.2	41.9	6.1	41.3	6.7	0.001 *	0.008 *	0.769

PPG = pre-perimetric glaucoma; PG = perimetric glaucoma; SD = standard deviation; MD = mean defect; sLV = square loss variance; ONH = optic nerve head; CDR = cup–disc ratio; GCC = ganglion cell complex; RNFL = retinal nerve fiber layer; RPC = radial peripapillary capillary; VDms = vessel density of macular superficial vascular complex; VDmd = vessel density of macular deep vascular complex. * statistically significant (*p* < 0.01).

**Table 3:** Results of receiver operating characteristic analysis between control and pre-perimetric glaucoma group.

Parameters	AUC	SE	*p* Value	Best Cut-Off	Se	Sp	Se at 80% Sp	Se at 90% Sp
**Pulsar perimetry**								
MD (src)	0.642	0.048	0.003 *	>−0.7	87.5	35.2	30.0	25.0
sLV (src)	0.673	0.046	<0.001 *	>1.6	85.0	44.8	43.0	20.0
**OCT-ONH analysis**								
Vertical CDR	0.652	0.050	0.002 *	>0.59	50.0	80.7	50.0	22.8
Horizontal CDR	0.566	0.051	0.195	>0.65	55.0	57.2	31.3	16.3
Rim. Area (mm^2^)	0.623	0.048	0.010	≤1.25	67.5	58.6	37.5	8.1
**OCT-GCC thickness (μm)**								
Whole Image	0.836	0.033	<0.001 *	≤95	77.5	73.1	65.0	55.0
Superior	0.860	0.030	<0.001 *	≤96	90.0	66.2	67.5	62.5
Inferior	0.822	0.035	<0.001 *	≤92	60.0	86.9	62.5	50.0
**OCTA (Disc)-RNFL thickness (μm)**								
Peripapillary	0.780	0.046	<0.001 *	≤109	77.5	71.0	66.7	47.5
Superior-Hemi	0.768	0.044	<0.001 *	≤109	75.0	71.0	55.0	44.7
Inferior-Hemi	0.739	0.050	<0.001 *	≤106	67.5	77.9	61.9	41.3
**OCTA (Macular)-Retina thickness (μm)**								
Whole Image	0.814	0.036	<0.001 *	≤276	77.5	71.0	62.5	48.8
Superior-Hemi	0.817	0.034	<0.001 *	≤275	65.0	82.1	66.5	41.7
Inferior-Hemi	0.794	0.040	<0.001 *	≤270	70.0	75.9	63.8	45.0
Fovea	0.615	0.047	0.014	≤242	60.0	64.1	27.5	10.0
ParaFovea	0.736	0.043	<0.001 *	≤313	75.0	62.8	49.5	30.8
PeriFovea	0.803	0.038	<0.001 *	≤276	80.0	65.5	62.5	50.6
**OCTA (Disc)-RPC (%)**								
Whole Image	0.791	0.041	<0.001 *	≤47.4	70	80.7	70	51.3
Inside Disc	0.543	0.051	0.397	≤51.9	62.5	51.0	22.5	12.5
Peripapillary	0.781	0.043	<0.001 *	≤48.8	55	87.6	60	50.4
Superior-Hemi	0.749	0.046	<0.001 *	≤49.0	52.5	89.7	55.6	51.3
Inferior-Hemi	0.775	0.043	<0.001 *	≤50.2	70	74.5	62.5	40
**OCTA (Macular)-VDms (%)**								
Whole Image	0.684	0.046	<0.001 *	≤47.2	70.0	62.8	38.1	24.4
Superior-Hemi	0.678	0.047	<0.001 *	≤45.7	57.5	77.9	50.0	16.3
Inferior-Hemi	0.686	0.046	<0.001 *	≤47.7	80.0	55.9	32.5	25.6
Fovea	0.635	0.048	0.005 *	≤20.6	77.5	47.6	27.5	22.5
ParaFovea	0.642	0.046	0.002 *	≤51.1	90.0	40.0	27.5	12.5
PeriFovea	0.681	0.047	<0.001 *	≤47.3	65.0	70.3	37.5	27.5
**OCTA (Macular)-VDmd (%)**								
Whole Image	0.673	0.050	<0.001 *	≤40.0	52.5	79.3	50.0	27.5
Superior-Hemi	0.655	0.052	0.003 *	≤42.0	60.0	69.7	42.5	31.3
Inferior-Hemi	0.682	0.048	<0.001 *	≤43.8	75.0	55.2	47.5	27.5
Fovea	0.622	0.045	0.007 *	≤37.5	82.5	44.1	27.5	7.5
ParaFovea	0.646	0.050	0.004 *	≤52.9	85.0	40.7	37.5	25.0
PeriFovea	0.682	0.049	<0.001 *	≤46.2	85.0	46.9	45.0	27.5

AUC = area under the curve; SE = standard error; Se = sensitivity; Sp = specificity; MD = mean defect; sLV = square loss variance; ONH = optic nerve head; CDR = cup–disc ratio; GCC = ganglion cell complex; RNFL = retinal nerve fiber layer; RPC = radial peripapillary capillary; VDms = vessel density of macular superficial vascular complex; VDmd = vessel density of macular deep vascular complex. * statistically significant (*p* < 0.01).

**Table 4:** Results of receiver operating characteristic analysis between control and perimetric glaucoma group.

Parameters	AUC	SE	*p* Value	Best Cut-Off	Se	Sp	Se at 80% Sp	Se at 90% Sp
**Pulsar perimetry**								
MD (src)	0.885	0.033	<0.001 *	>0.9	100.0	64.8	76.5	58.8
sLV (src)	0.810	0.056	<0.001 *	>2.2	76.5	74.5	68.2	50.0
**OCT-ONH analysis**								
Vertical CDR	0.631	0.077	0.087	>0.51	70.6	57.2	41.2	23.5
Horizontal CDR	0.558	0.075	0.439	>0.52	94.1	25.5	29.4	17.7
Rim. Area (mm^2^)	0.681	0.080	0.023	≤1.14	64.7	71.7	47.1	35.3
**OCT-GCC thickness (μm)**								
Whole Image	0.847	0.056	<0.001 *	≤95	88.2	70.3	70.6	52.9
Superior	0.768	0.071	<0.001 *	≤96	82.4	66.2	52.9	41.2
Inferior	0.832	0.051	<0.001 *	≤95	82.4	64.8	58.8	52.9
**OCTA (Disc)-RNFL thickness (μm)**								
Peripapillary	0.841	0.057	<0.001 *	≤104	76.5	87.6	76.5	63.2
Superior-Hemi	0.856	0.052	<0.001 *	≤105	82.4	80.0	82.4	62.5
Inferior-Hemi	0.783	0.073	<0.001 *	≤107	76.5	75.2	70.6	47.1
**OCTA (Macular)-Retina thickness (μm)**								
Whole Image	0.825	0.047	<0.001 *	≤281	94.1	58.6	57.1	52.9
Superior-Hemi	0.801	0.046	<0.001 *	≤285	100.0	51.7	52.9	45.1
Inferior-Hemi	0.827	0.055	<0.001 *	≤262	58.8	93.8	67.7	58.8
Fovea	0.553	0.067	0.432	≤260	94.1	28.3	19.6	8.8
ParaFovea	0.730	0.052	<0.001 *	≤321	100.0	44.1	47.1	17.7
PeriFovea	0.833	0.046	<0.001 *	≤280	100.0	53.1	58.8	52.9
**OCTA (Disc)-RPC (%)**								
Whole Image	0.809	0.063	<0.001 *	≤48.3	82.4	66.9	61.8	52.9
Inside Disc	0.611	0.083	0.184	≤43.8	35.3	89.7	41.2	29.4
Peripapillary	0.752	0.083	0.002 *	≤48.2	58.8	91.0	64.7	58.8
Superior-Hemi	0.734	0.088	0.008 *	≤47.2	64.7	94.5	64.7	64.7
Inferior-Hemi	0.697	0.086	0.022	≤47.4	47.1	94.5	58.8	47.1
**OCTA (Macular)-VDms (%)**								
Whole Image	0.715	0.077	0.005 *	≤46.4	70.6	70.3	52.9	35.3
Superior-Hemi	0.716	0.076	0.005 *	≤46.3	70.6	72.4	47.1	29.4
Inferior-Hemi	0.714	0.075	0.004 *	≤46.8	76.5	64.8	47.1	35.3
Fovea	0.714	0.064	<0.001 *	≤20.4	88.2	47.6	47.1	29.4
ParaFovea	0.731	0.076	0.002 *	≤46.3	70.6	72.4	58.8	47.1
PeriFovea	0.714	0.075	0.004 *	≤48.2	76.5	63.5	41.2	41.2
**OCTA (Macular)-VDmd (%)**								
Whole Image	0.669	0.081	0.037	≤39.0	52.9	86.2	52.9	47.1
Superior-Hemi	0.636	0.082	0.096	≤39.6	47.1	83.5	47.1	29.4
Inferior-Hemi	0.686	0.080	0.020	≤38.3	52.9	89.0	52.9	47.1
Fovea	0.721	0.056	<0.001 *	≤32.8	70.6	71.7	47.1	11.8
ParaFovea	0.609	0.084	0.194	≤47.0	47.1	85.5	47.1	23.5
PeriFovea	0.679	0.076	0.018	≤38.3	47.1	89.0	47.1	38.2

AUC = area under the curve; SE = standard error; Se = sensitivity; Sp = specificity; MD = mean defect; sLV = square loss variance; ONH = optic nerve head; CDR = cup–disc ratio; GCC = ganglion cell complex; RNFL = retinal nerve fiber layer; RPC = radial peripapillary capillary; VDms = vessel density of macular superficial vascular complex; VDmd = vessel density of macular deep vascular complex. * statistically significant (*p* < 0.01).

**Table 5:** Correlation matrix of generalized structural and vascular parameters of OCT and OCTA and functional parameters.

Parameters	ONH- Rim. Area	GCC-Whole	RNFL- Peripapillary	Retina-Whole	RPC-Whole	VDms-Whole	VDmd-Whole
GCC-Whole	0.132(0.061)	1					
RNFL-Peripapillary	0.274(<0.001 *)	0.427(<0.001 *)	1				
Retina-Whole	0.067(0.346)	0.795(<0.001 *)	0.345(<0.001 *)	1			
RPC-Whole	0.226(0.001 *)	0.205(0.003 *)	0.337(<0.001 *)	0.166(0.018)	1		
VDms-Whole	0.049(0.488)	0.386(<0.001 *)	0.175(0.013)	0.307(<0.001 *)	0.226(<0.001 *)	1	
VDmd-Whole	−0.014(0.839)	0.180(0.010)	0.018(0.802)	0.141(0.046)	0.159(0.024)	0.525(<0.001 *)	1
PP-MD	0.031(0.657)	−0.146(0.039)	−0.153(0.030)	−0.169(0.016)	−0.187(0.008 *)	−0.223(0.001 *)	−0.217(0.002 *)
SAP-MD	−0.064(0.365)	−0.186(0.008 *)	−0.156(0.026)	−0.145(0.039)	−0.153(0.030)	−0.217(0.002 *)	−0.144(0.041)

ONH = optic nerve head; GCC = ganglion cell complex; RNFL = retinal nerve fiber layer; RPC = radial peripapillary capillary; VDms = vessel density of macular superficial vascular complex; VDmd = vessel density of macular deep vascular complex; PP = Pulsar perimetry; MD = mean defect; SAP = standard actometry perimetry. Data are expressed as Pearson’s r (*p* value). * statistically significant (*p* < 0.01).

## Data Availability

The data presented in this study are available on request from the corresponding author.
